# Rectal Squamous Cell Cancer

**DOI:** 10.7759/cureus.15133

**Published:** 2021-05-20

**Authors:** Ikramamul L Nibir, Awana N Chowdhury, John W Bollinger

**Affiliations:** 1 College of Osteopathic Medicine, Lake Erie College of Osteopathic Medicine, Bradenton, USA; 2 College of Osteopathic Medicine, Nova Southeastern University, Fort Lauderdale, USA; 3 Radiation Oncology, AdventHealth Florida, Sebring, USA

**Keywords:** squamous cell carcinoma (scc), human papillomavirus (hpv), general radiation oncology, distal rectal cancer, rectal polyps, colorectal cancer, concomitant chemoradiotherapy

## Abstract

Squamous cell carcinoma occurring in the rectum is one of the rare malignancies that has been discovered. Most squamous cell carcinomas that surface in the gastrointestinal tract tend to occur in either the esophagus or the anal canal. However, the rare incidence of rectal squamous cell carcinomas has raised quite a few questions on the hypothetical etiologies, prognosis, and optimal treatment sequence of such a disease course in modern medicine. In this report, we present the case of a 63-year-old gentleman who came to the clinic with change in bowel habits such as constipation and bright red blood in his stool. Colonoscopy revealed a 4.1 cm polyp in the distal rectum, which upon biopsy was confirmed to be a well-differentiated keratinizing squamous cell carcinoma. This case allows us to engage in discussions over potential etiologies and current treatment management for such a rare malignancy.

## Introduction

The very first rectal squamous cell cancer was discovered in 1933, and since then, the incidence of this malignancy has been reported to be 0.10 to 0.25 per 1,000 colorectal cancers [1]. While adenocarcinomas make up over 90% of colorectal cancers, squamous cell carcinoma remains rarer than other types of colorectal malignancies such as carcinoid tumors, stromal tumors, lymphomas, and leiomyosarcomas. However, when squamous cell carcinomas do occur, the most common location tend to be in the rectum followed by the right colon. We present this case of a 63-year-old gentleman who presented with hematochezia and constipation to discuss the potential sources of the disease and what treatment options are considered optimal for better patient outcomes.

## Case presentation

**Figure 1:**
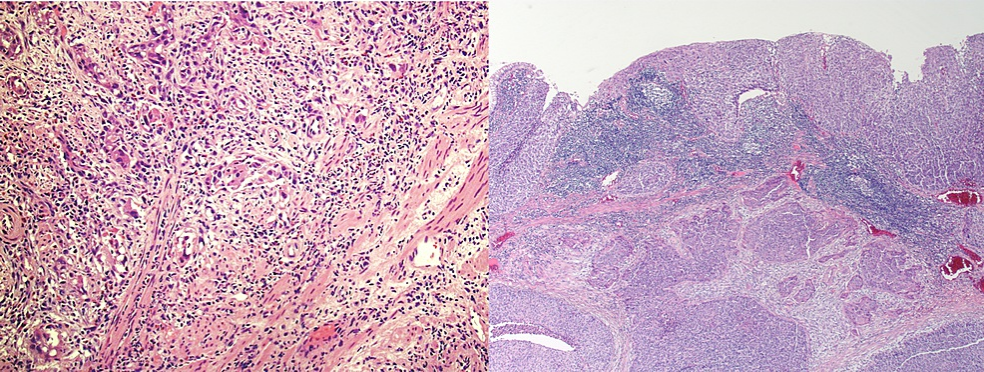
Biopsy of rectal squamous cell carcinoma.

A 63-year-old male presented to an outpatient clinic with two different episodes of hematochezia and worsening constipation for three months. He reported no significant weight loss, nausea, vomiting, diarrhea, changes in stool caliber, loss of appetite, or anal intercourse. His vitals were unremarkable, the abdomen was soft, non-tender, without hepatosplenomegaly, and there were no appreciable masses on rectal examination. Hemoccult test was positive, hemoglobin level was 13.7 g/dL, and hematocrit was 39.4%. Family history was remarkable for a history of colon cancer in his mother and lung cancer in his father. The patient had a colonoscopy seven years ago which was unremarkable. However, the latest colonoscopy that was ordered after presenting with alarming symptoms revealed a large polyp in the rectum and another polyp in the transverse colon along with findings of diverticulosis and internal hemorrhoids. Biopsies of the polyps revealed that the transverse polyp was tubular adenoma, and after confirming with a second opinion, the rectal polyp was determined to be a well-differentiated keratinizing squamous cell carcinoma (Figure 1).

**Figure 2:**
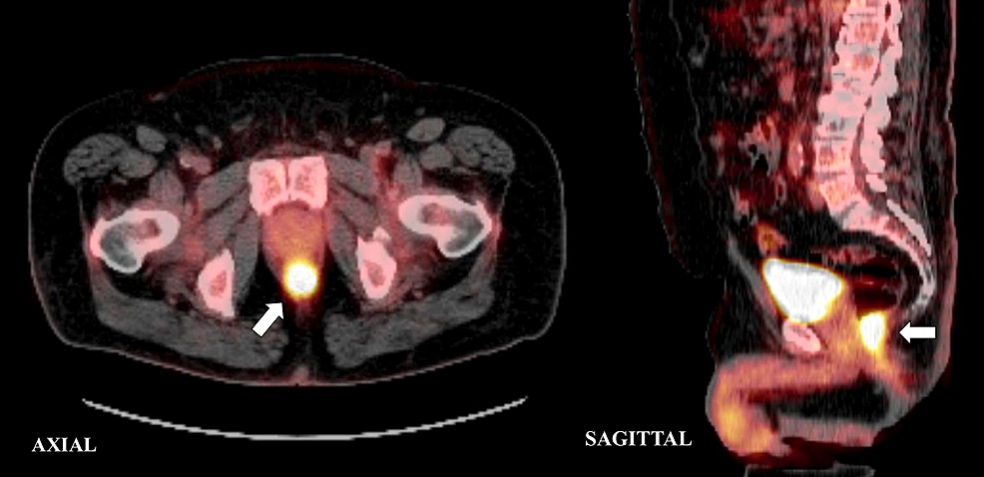
Axial and sagittal view of PET-CT scan demonstrating increased FDG uptake in the rectum. FDG: ^18^F-fluoro-2-deoxyglucose; PET-CT: positron emission tomography-computed tomography

**Figure 3:**
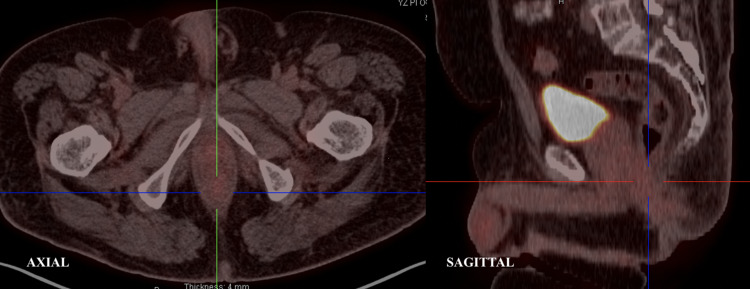
Axial and sagittal view of post-chemoradiotherapy PET-CT. PET-CT: positron emission tomography-computed tomography

Following the biopsies, a positron emission tomography-computed tomography (PET-CT) was performed (Figure 2), which revealed a 4.1 cm hypermetabolic mass in the distal rectum with maximum standard uptake value (SUV) of 13.5 with no evidence of lymphadenopathy or any distant metastatic disease. Furthermore, the case was presented at multidisciplinary rounds and a decision to proceed with definitive chemoradiation therapy was made. It was agreed that the rectal squamous cell cancer should be treated the same way as anal squamous cell carcinoma. The patient was planned for a CT simulation for radiation therapy arrangement. He was initially prescribed 56 gray (Gy) in 28 fractions targeting the rectal mass and 42 Gy in 28 fractions towards the pelvic and inguinal nodes through intensity-modulated radiation therapy, along with continuous infusion of mitomycin and 5-fluorouracil per the National Comprehensive Cancer Network guidelines for rectal cancer. However, due to excessive radiation proctitis and skin toxicity, treatment was stopped at 48 Gy in 24 fractions to the primary cancer and 36 Gy in 24 fractions to the elective lymph nodes. Furthermore, a PET-CT (Figure 3) performed almost over two months after finishing his treatment revealed near complete resolution of the anal mass with an SUV max of 2.8 with no local or distal metastasis. In addition, repeat colonoscopy visualized no tumors and biopsies only confirmed slight inflammation. Currently, the patient is doing well and is being followed regularly.

## Discussion

Rectal squamous cell cancer is a rare entity, nevertheless, there have been many theories regarding the pathophysiology of the disease [2-4]. For example, one theory suggests that inflammation secondary to inflammatory bowel disease, infection, or radiation generates squamous metaplasia resulting in squamous carcinoma [2]. In our patient, this hypothesis holds little value because while his medical history did consist of diverticulosis, he did not have a history of ulcerative colitis, Crohn’s disease, serious infections, or any exposure to radiation prior to diagnosis. Furthermore, another theory that resonates more with our case is the understanding that squamous differentiation can result from preexisting adenomas or adenocarcinomas. This hypothesis is usually validated by histological reviews that suggest squamous differentiation in adenomas in other areas of the colon [3]. Our patient had evidence of polyps in the transverse colon as revealed by the PET-CT scan, which opens up the idea that squamous cell carcinoma of the rectum could potentially be a differentiated adenocarcinoma.

Another association that is not well established but definitely worth noting is the association of rectal squamous cell cancer with human papillomavirus (HPV). There are numerous cases reported of HPV resulting in squamous cell cancers of skin, oral, vaginal, penile, esophageal, and anal canal. Kinjo et al. in 2003 was the first one to study the effects of HPV transfection into adenocarcinoma cells in the colon and lungs. The results showed a clear relationship between HPV transfection and squamous metaplasia of colonic adenocarcinoma cells [4]. This reputable association of HPV with squamous cell cancers implored us to immunohistochemically stain for high-risk HPV p16. The staining results returned positive for high-risk HPV p16 strain yielding a strong potential etiology of squamous cell carcinoma of the rectum.

The treatment of rectal squamous cell cancer is yet to be standardized because of its infrequency. Though, recent cases of rectal squamous cell cancer have primarily been treated with the same protocols as anal squamous cell cancer because chemoradiotherapy has provided better outcomes in patients when compared to surgical resection followed by adjuvant chemotherapy or radiotherapy. Guerra et al. provided extensive data on treatment for rectal squamous cell carcinoma cases from 1933 to 2016, concluding overall survival of 86% for chemoradiotherapy group versus 48% for the traditional surgery group [5]. Moreover, Song et al. concluded with their analysis of a small cohort that chemoradiation therapy provides better disease-related outcomes, sphincter preservation, and morbidity profiles [6]. Lastly, Kommalapati et al. retrospectively analyzed 3,405 cases of squamous cell cancer of the rectum between 2004 and 2015 from the National Cancer Database. It was recognized in the study that outcomes of rectal squamous cell cancer were dependent upon age, sex, comorbidity score, and therapy received. Patients in stages I-III who received chemoradiation therapy alone had 108 months of overall survival, and patients who received surgery alone had 76 months of overall survival. Furthermore, no such difference in overall survival was noted in groups that received surgery with adjuvant chemoradiation therapy [7].

## Conclusions

Squamous cell carcinoma of the gastrointestinal tract most commonly occurs in the esophagus or anal canal, but the exceedingly rare cases of rectal squamous cell carcinomas are evident enough to implore the scientific community to investigate further. While the pathogenesis of the disease still remains unclear, the association with HPV seems significant. Treatment protocols from retrospective studies shows great promise for chemoradiation therapy as the mainstay treatment for better patient health outcomes. Further research is required to delve into the pathophysiology of the disease and establish first-line treatment practice.
